# Could high-concentration rifampicin kill rifampicin-resistant *M. tuberculosis*? Rifampicin MIC test in rifampicin-resistant isolates from patients with osteoarticular tuberculosis

**DOI:** 10.1186/s13018-014-0124-1

**Published:** 2014-12-03

**Authors:** Zehua Zhang, Fei Dai, Fei Luo, Min Zhong, Zhenggu Huang, Tianyong Hou, Jianzhong Xu

**Affiliations:** Department of Orthopaedics, Southwest Hospital, Third Military Medical University, Chongqing, 400038 China; Department of Clinical Laboratory Medicine, Chongqing Infectious Diseases Medical Center, Chongqing, 400038 China

**Keywords:** Rifampicin, Drug resistance, Osteoarticular tuberculosis, rpoB gene

## Abstract

**Purpose:**

Several studies have shown that the intralesional concentration of rifampicin in osteoarticular tuberculosis is typically at a subtherapeutic level. Sustained or controlled release by novel drug delivery systems has been investigated to maintain an effective rifampicin concentration, but the local administration of rifampicin remains controversial. Additionally, it is still unclear whether high-dose rifampicin could kill rifampicin-resistant *Mycobacterium tuberculosis*. The aim of this study was to assess the in vitro killing effect of high-concentration rifampicin on rifampicin-resistant *M. tuberculosis* isolated from patients with osteoarticular tuberculosis.

**Methods:**

A set of 18 rifampicin-resistant *M. tuberculosis* isolates by the BACT/MGIT 960 system from patients with osteoarticular tuberculosis was collected for further study. The detection of rpoB gene mutations was performed using non-fluorescent, low-density DNA microarrays to determine the resistant mechanism. Following secondary culture, susceptibility to gradient concentrations of rifampicin (2 to 256 μg/ml) was tested; these concentrations are attainable for prolonged periods of local chemotherapy. The relationship between microbial killing by high-dose rifampicin and rpoB gene mutations was analyzed.

**Results:**

Mutations in the rifampicin resistance-determining region (RRDR) of the rpoB gene were identified in 17 isolates (94.4%); one strain exhibited no mutations in this region. The most prevalent mutation sites were in codons 531 (55.56%), 516 (16.67%), 526 (11.11%), and 513 (11.11%). Isolates with mutations in the rpoB gene were highly resistant to rifampicin, 11 of which had minimal inhibitory concentrations (MICs) exceeding 256 μg/ml (not determined). The MICs for the remaining seven resistant isolates were between 32 and 256 μg/ml. Particularly in less rifampicin-resistant *M. tuberculosis* strains, growth was inhibited at high concentrations.

**Conclusion:**

Increasing the rifampicin concentration may optimize this drug’s antituberculous effect, even against some rifampicin-resistant isolates, if systemic and local toxic effects can be minimized.

## Introduction

One-third of the world’s population is infected with *Mycobacterium tuberculosis*. There were almost 9 million new cases in 2011 and 1.4 million tuberculosis (TB) deaths, with China accounting for nearly 17% of the worldwide TB burden, second only to India in the number of TB patients [1]. Extra-pulmonary TB was usually non-contagious, and it did not attract much public attention. Unfortunately, the incidence of osteoarticular tuberculosis has risen concurrently with that of pulmonary tuberculosis. Osteoarticular tuberculosis condition requires more attention, especially considering the large number of affected people and its resultant disability and mortality [2]. Additionally, multidrug-resistant tuberculosis (MDR-TB) is on the rise worldwide, posing a significant threat to the world’s population.

Since rifampicin was introduced as an antituberculous agent in 1963, it has been the cornerstone of drug regimens for the treatment of tuberculosis. Studies have demonstrated variable bioavailability and low plasma concentrations of rifampicin [3], as well as intralesional concentrations of rifampicin and pyrazinamide in osteoarticular tuberculosis at mostly subtherapeutic levels [4]. Therefore, rifampicin has been associated with clinical failure and drug resistance.

In some drug resistance test by absolute concentration method, low-level resistance of *M. tuberculosis* to rifampicin was overcome by an increase in the drug concentration from 50 to 250 μg/ml, implying that the drug’s antituberculous effect can be optimized by increasing the dose [5]. The local administration of rifampicin may be an effective clinical approach to treat osteoarticular tuberculosis. Our study in vitro aimed to determine the killing effect of high-concentration rifampicin on rifampicin-resistant isolates from patients with osteoarticular tuberculosis.

## Materials and methods

### Bacterial strain isolation

A total of 198 patients with osteoarticular tuberculosis were admitted to Southwest Hospital (Chongqing, China) and the Chongqing Infectious Diseases Medical Center (Chongqing, China) from 1 December 2006 to 1 December 2011. The study was approved by the Ethics Committee of Southwest Hospital. Written informed consent was obtained from all of the patients. Cold abscess and caseous tissue necrosis samples were collected during surgery. These specimens were processed according to standard protocols. The same amount of each concentrated sample (0.5 ml) was inoculated into vials for use with the BacT/ALERT 3D Microbial Detection System (bioMerieux, France) containing modified Middlebrook 7H9 broth supplemented with an antibiotic. All mycobacterial cultures were incubated at 37°C. The detection of mycobacterial growth by the BacT/ALERT 3D Microbial Detection System is based on colorimetric detection of carbon dioxide, and the cultures are continuously monitored by the automated system. Mycobacterial growth was also verified by Ziehl-Neelsen staining and microscopy [6-9]. Drug susceptibility testing was performed on the first isolate from each patient using the absolute concentration method on Löwenstein-Jensen (L-J) medium. This testing included 11 first- and second-line drugs. For all drugs, except pyrazinamide, the following critical concentrations were used: 50 and 250 μg/ml rifampicin, 1 and 10 μg/ml isoniazid, 5 and 50 μg/ml ethambutol, 10 and 100 μg/ml streptomycin, 5 and 50 μg/ml levofloxacin, 1 and 10 μg/ml PAS, 25 and 100 μg/ml protionamide, 0.1 and 1 μg/ml pasiniazid, 50 and 250 μg/ml rifapentine, 10 and 100 μg/ml capreomycin, and 10 and 100 μg/ml amikacin. The L-J slants were inspected weekly for growth over a total of 8 weeks [10].

A set of 18 rifampicin-resistant *M. tuberculosis* strains were obtained and recovered using the BacT/ALERT 3D Microbial Detection System. The standard *M. tuberculosis* strain H37Rv (from the National Institutes for Food and Drug Control of China) was employed as a control. Each culture was used for susceptibility testing within 3 days after the instrument detected a positive signal.

### Detection of rpoB mutations using a DNA probe array

The template DNA for molecular sequencing was prepared by heat-killing mycobacteria at 95°C for 30 min, sonicating the bacteria at room temperature for 25 min, and centrifuging the sample at 5,000 rpm for 1 min. The supernatant was then stored at −20°C until needed.

A commercial PCR-based reverse hybridization line probe assay (*M. tuberculosis* Drug Resistance Detection Array Kit; CapitalBio Corporation, China) using biotinylated primers was performed manually according to the manufacturer’s instructions. This assay consists of specific oligonucleotides immobilized at known locations on membrane strips and hybridized under strictly controlled conditions with a biotin-labeled PCR product (Table 1).

**Table 1:** **Schematic diagram of the DNA probe array for rpoB detection**

	**1**	**2**	**3**	**4**	**5**	**6**	**7**	**8**	**9**	**10**
1	QC					EC				
2	BC					rpoB-IC				
3	*Mycobacterium*					*M. tuberculosis*				
4	511-WT					511 T → C				
5	513-WT					513 C → A				
6	516-WT					513 A → C				
7	533-WT					533 T → C				
8	531-WT					531 C → T				
9	526-WT					531 C → G				
10	526 C → T					526 C → G				
11	526 A → T					526 A → G				
12	516 A → T					516 G → T				
13	516 A → G					NC				
14	EC					QC				

All probes (511, 513, 516, 531, 526, and 533) were immobilized horizontally five times. QC, quality control; EC, external control; BC, blank control; NC, negative control; IC, internal control; WT, wild-type.

The fluorescent signal emitted by the target bound to the array was detected at a pixel resolution of 3 μm using a LuxScan™ 10 K Microarray Scanner (CapitalBio). Probe array cell intensities, nucleotide base calls, and mutation determinations and reports were generated using GeneChip software (CapitalBio) [11-14].

### Rifampicin susceptibility testing of rifampicin-resistant *M. tuberculosis*

A stock solution of rifampicin was prepared by dissolving 80 mg of rifampicin in 1 ml of DMSO to attain a final concentration of 80 mg/ml. This solution was then aliquoted and stored at −20°C. Eight dilutions (bioMerieux) were prepared by diluting the rifampicin stock solution with modified Middlebrook 7H9 to achieve the desired concentrations by the broth microdilution method (serial twofold dilutions of rifampicin, ranging from 2 to 256 mg/ml). Culture vials containing antibiotics, as well as one drug-free control vial, were inoculated to reach a final concentration of approximately 3 × 10^5^ to 3 × 10^6^ cfu/ml. All culture vials were incubated in the BacT/ALERT 3D Microbial Detection System instrument and continuously monitored until the results, indicating susceptibility or resistance, were automatically interpreted and reported by the system based on predefined algorithms. These algorithms compared growth in the drug-containing tubes to that in the growth control tube. Next, 0.5 ml of MB/BacT Antibiotic Supplement (bioMerieux) was added to the BacT/ALERT culture vials. These vials were tested daily for up to 12 days, which is the maximum incubation time for antimycobacterial susceptibility testing. Ziehl-Neelsen staining was used to confirm the presence of acid-fast bacilli in all positive media.

### Statistical analysis

The results were analyzed using Wilcoxon’s signed rank test. *P* values of less than 0.05 were considered statistically significant. All analyses were carried out using SPSS 13.0 software.

## Results

**Figure 1:**
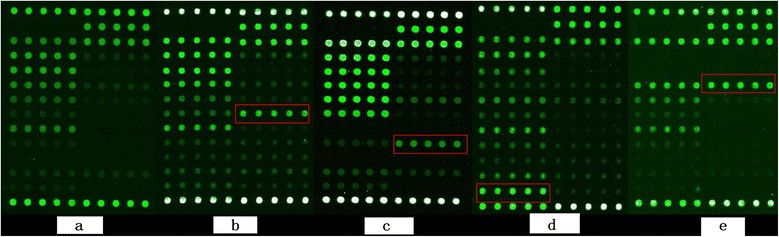
**Detection of** ***M. tuberculosis*** **rpoB mutants using a DNA probe array. (a)** Wild-type without high hybridization signal of the mutated probe as control. **(b)** RMP resistance with the rpoB 531 (TCG → TTG) mutation (higher hybridization signal, red rectangle). **(c)** RMP resistance test: rpoB 526 (CAC → GAC) mutation (red rectangle). **(d)** RMP resistance with the rpoB 516 (GAC → GGC) mutation (red rectangle). **(e)** RMP resistance with the rpoB 531 (CAA → CCA) mutation (red rectangle).

In this study, mutations in the rifampicin resistance-determining region (RRDR) of the rpoB gene were identified in 17 (94.4%) of the 18 rifampicin-resistant isolates, with one rifampicin-resistant strain exhibiting no mutations in this region. The most prevalent mutation sites were in codons 531 (55.56%), 516 (16.67%), 526 (11.11%), and 513 (11.11%) (Figure 1). High-level rifampicin-resistant strains (resistant to 250 μg/ml) exhibited a higher mutation frequency at 531-Ser than low-level rifampicin-resistant strains (resistant to 50 μg/ml) (*P* < 0.05). The growth of the H37Rv strain was inhibited at rifampicin concentrations ≤0.25 μg/ml, indicating drug susceptibility.

The relationship between the degree of resistance to rifampicin and the mutation site was characterized by the minimal inhibitory concentration (MIC) test. Isolates with mutations in the rpoB gene were highly resistant to rifampicin, 11 of which had MICs exceeding 256 μg/ml (not determined), and 81.81% (9/11) had mutation in codons 531. The MICs for the remaining seven resistant isolates were between 32 and 256 μg/ml, and only 14.29% (1/7) had mutation in codons 531. Particularly in low-level rifampicin-resistant *M. tuberculosis* strains, growth was inhibited at high concentrations (Table 2).

**Table 2:** ***M. tuberculosis*** **rpoB wild-type and mutant isolates analyzed using the rpoB probe array**

**Clinical isolates**	**Resistance phenotype**	**MIC (μg/ml)**	**Mutated codon(s)**
1	L	32	WT
2	L	64	516 A → G
3	L	64	516 A → G
4	L	128	513 A → C
5	H	128	526 C → G
6	H	256	513 A → C
7	H	256	531 C → T
8	H	ND	531 C → T
9	H	ND	531 C → T
10	H	ND	531 C → T
11	H	ND	531 C → T
12	H	ND	531 C → T
13	H	ND	531 C → T
14	H	ND	531 C → T
15	H	ND	531 C → T
16	H	ND	531 C → G
17	H	ND	526 C → T
18	H	ND	516 A → G
H37Rv	S	0.25	WT

S, susceptible to rifampicin; L, low-level resistance to rifampicin (resistant to 50 μg/ml rifampicin); H, high-level resistance to rifampicin (resistant to 250 μg/ml rifampicin); ND, not determined; WT, wild-type.

## Discussion

Rifampicin is a key component of standard antituberculosis regimens. This drug exhibits a significant early bactericidal effect on metabolically active *M. tuberculosis*. However, data suggest that the standard rifampicin dose is probably at the lower limit of optimal efficacy. The currently applied dose of 10 mg of drug per kilogram of body weight may only result in low plasma concentrations of rifampicin [3]. Several studies have also shown that the intralesional concentration of rifampicin in osteoarticular tuberculosis is mostly subtherapeutic. Jutte and colleagues found that the penetration of isoniazid in tuberculous pleural effusion and psoas abscess was always sufficient, while the penetration of rifampicin was mostly below the desired ratio, and that of pyrazinamide was ten times too low on average [4]. Therefore, rifampicin may potentially fail in patients with active disease and may even contribute to the increasing resistance to antituberculosis drugs. Unfortunately, the causes of poor or variable bioavailability of rifampicin are not clearly understood. This problem is particularly evident when rifampicin is present in antituberculous fixed-dose combination products, which is a matter of serious concern. The enhanced decomposition of rifampicin in the presence of isoniazid in the stomach after ingestion may be a key reason for the problem. Crystalline changes in the drug and inadequate blood supply to the tuberculous lesion are also cited as the principal reasons [15].

The mechanisms underlying drug resistance are often multifaceted and may include not only chromosomal mutations but also induction or the presence of efflux pumps, and even antagonism of the component drugs in combination therapy. Mutations in the rpoB gene, encoding the β subunit of the bacterial RNA polymerase, have been strongly associated with rifampicin (RMP)-resistant phenotypes in multiple study populations. rpoB mutations are most common in an 81-bp region called the RRDR. Up to 90% of RMP-resistant strains carry RRDR mutations at codons 516, 526, or 531 [16]. In this study, the majority (94.44%) of rifampicin-resistant *M. tuberculosis* isolates were found to contain point mutations at codons 531 (55.56%), 516 (16.67%), 526 (11.11%), or 513 (11.11%), which are located in the core region of the rpoB gene. Codons 531 and 526 are the most common sites of nucleotide substitutions worldwide; the difference between the worldwide average and our data may be attributed to sampling error and geographical genetic differences in RMP-resistant *M. tuberculosis* strains [17,18].

The use of certain antituberculous drugs for the treatment of low-level rifampicin-resistant *M. tuberculosis* remains controversial. To assess the feasibility of isoniazid for the treatment of isoniazid (INH)-resistant tuberculosis, Schaaf and colleagues measured the minimal inhibitory concentration of isoniazid in isoniazid-resistant *M. tuberculosis* isolates obtained from children. They found that for 80% of the isoniazid-resistant strains for which the MIC was relatively low, high-dose INH at 15–20 mg/kg/day could still improve treatment [19]. It is unclear whether higher doses of RMP could similarly increase the antituberculous activity of RMP. Gumbo and coworkers demonstrated that rifampicin’s microbial killing is concentration-dependent and is linked to the ratio of the area under the concentration-time curve to the MIC. The suppression of resistance is associated with the free peak concentration (Cmax)-to-MIC ratio and not to the duration for which the rifampicin concentration is above the MIC. In a previous study, rifampicin was shown to prevent resistance to itself at a free Cmax/MIC ratio of ≥175 [20], implying that higher doses of rifampicin than those currently employed would optimize its microbial killing effect, if tolerated by patients. Our results show that a portion of rifampicin-resistant isolates (38.89%) could be killed by higher concentrations of rifampicin. The reason for this finding is unclear but may be related to saturable efflux transporter proteins. More specifically, the concentration-dependent effects of rifampicin may be partly explained by what appears to be enhanced rifampicin entry at higher concentrations in the bacillary milieu [18,20]. The strains with higher MICs were found to have a higher frequency of the 531 codon mutation than strains with lower MICs (*P* < 0.05). These results suggest that mutations in the rpoB gene are mostly, but not necessarily, associated with *M. tuberculosis* rifampicin resistance and that the sites of the mutations in the rpoB gene may affect the level of resistance to rifampicin.

In the light of these results, local administration of rifampicin is worth considering to achieve higher concentrations. Rifampicin Injection USP was approved by the FDA on 29 October 1999. However, the clinical use of rifampicin injection in China is still uncommon compared to ingestion of rifampicin capsules. Rifampicin injection was the most widely available drug form when considering practicability of local chemotherapy. Following the development of sustained/controlled release techniques, an implantable drug delivery system for osteoarticular tuberculosis may have clinical applications in the future. However, the possibility of the selective enrichment of rifampicin-resistant bacteria and local toxic effects needs to be considered.

In summary, the results of the current study suggest that increasing the rifampicin concentration in tuberculous lesions may optimize the drug’s antituberculous effect, even for some rifampicin-resistant isolates, as long as systemic and local toxic effects are minimized. Further studies may be required to determine the microbial killing and resistance suppression ability of locally administered rifampicin.

## References

[1] **World Health Organization:** WHO report 2012 global tuberculosis control [EB/OL]**. Geneva: World Health Organization, 2012[2014-01-19.** http://www.who.int/tb/publications/global_report/gtbr12_main.pdf**.**

[2] Huang J, Shen M, Sun Y (2002). Characterization of rpoB mutations in rifampicin-resistant *Mycobacterium tuberculosis* isolated in China. Tuberculosis.

[3] van Crevel R, Alisjahbana B, de Lange WC, Borst F, Danusantoso H, van der Meer JW, Burger D, Nelwan RH (2002). Low plasma concentrations of rifampicin in tuberculosis patients in Indonesia. Int J Tuberc Lung Dis.

[4] Jutte PC, Rutgers SR, Van Altena R, Uges DR, Van Horn JR (2004). Penetration of isoniazid, rifampicin and pyrazinamide in tuberculous pleural effusion and psoas abscess. Int J Tuberc Lung Dis.

[5] Li L, Zhang Z, Luo F, Xu J, Cheng P, Wu Z, Zhou Q, He Q, Dai F, Wang J, Zhang J (2012). Management of drug-resistant spinal tuberculosis with a combination of surgery and individualized chemotherapy: a retrospective analysis of 35 patients. Int Orthop.

[6] Carricajo A, Fonsale N, Vautrin AC, Aubert G (2001). Evaluation of BacT/Alert 3D liquid culture system for recovery of mycobacteria from clinical specimens using sodium dodecyl (lauryl) sulfate-NaOH decontamination. J Clin Microbiol.

[7] Garrigó M, Aragón LM, Alcaide F, Borrell S, Cardeñosa E, Galán JJ, Gonzalez-Martín J, Martin-Casabona N, Moreno C, Salvado M, Coll P (2007). Multicenter laboratory evaluation of the MB/BacT *Mycobacterium* detection system and the BACTEC MGIT 960 system in comparison with the BACTEC 460 TB system for susceptibility testing of *Mycobacterium tuberculosis*. J Clin Microbiol.

[8] Scarparo C, Ricordi P, Ruggiero G, Piccoli P (2004). Evaluation of the fully automated BACTEC MGIT 960 system for testing susceptibility of *Mycobacterium tuberculosis* to pyrazinamide, streptomycin, isoniazid, rifampin, and ethambutol and comparison with the radiometric BACTEC 460 TB. Method J Clin Microbiol.

[9] Werngren J, Klintz L, Hoffner SE (2006). Evaluation of a novel kit for use with the BacT/ALERT 3D system for drug susceptibility testing of *Mycobacterium tuberculosis*. J Clin Microbiol.

[10] Xu L, JZ X, XM L, BF G (2013). Drug susceptibility testing guided treatment for drug-resistant spinal tuberculosis: a retrospective analysis of 19 patients. Int Surg.

[11] Riska PF, Jacobs WR, Alland D (2000). Molecular determinants of drug resistance in tuberculosis. Int J Tuberc Lung Dis.

[12] Aragón LM, Navarro F, Heiser V, Garrigó M, Español M, Coll P (2006). Rapid detection of specific gene mutations associated with isoniazid or rifampicin resistance in *Mycobacterium tuberculosis* clinical isolates using non-fluorescent low-density DNA microarrays. J Antimicrob Chemother.

[13] Sougakoff W, Rodrigue M, Truffot-Pernot C, Renard M, Durin N, Szpytma M, Vachon R, Troesch A, Jarlier V (2004). Use of a high-density DNA probe array for detecting mutations involved in rifampicin resistance in *Mycobacterium tuberculosis*. Eur J Clin Microbiol Infect Dis.

[14] Zhang ZH, Li LT, Luo F, Cheng P, Wu F, Wu Z, Hou TY, Zhong M, Xu JZ (2012). Rapid and accurate detection of RMP-and INH-resistant *Mycobacterium tuberculosis* in spinal tuberculosis specimens by CapitalBio DNA microarray: a prospective validation study. BMC Infect Dis.

[15] Ellard GA, Fourie PB (1999). Rifampicin bioavailability: a review of its pharmacology and the chemotherapeutic necessity for ensuring optimal absorption. Int J Tuberc Lung Dis.

[16] Telenti A, Imboden P, Marchesi F, Lowrie D, Cole S, Colston MJ, Matter L, Schopfer K, Bodmer T (1993). Detection of rifampicin-resistance mutations in *Mycobacterium tuberculosis*. Lancet.

[17] Hwang HY, Chang CY, Chang LL, Chang SF, Chang YH, Chen YJ (2003). Characterization of rifampicin-resistant *Mycobacterium tuberculosis* in Taiwan. J Med Microbiol.

[18] Ohno H, Koga H, Kohno S, Tashiro T, Hara K (1996). Relationship between rifampin MICs for and rpoB mutations of *Mycobacterium tuberculosis* strains isolated in Japan. Antimicrob Agents Chemother.

[19] Schaaf HS, Victor TC, Engelke E, Brittle W, Marais BJ, Hesseling AC, van Helden PD, Donald PR (2006). Minimal inhibitory concentration of isoniazid in isoniazid-resistant *Mycobacterium tuberculosis* isolates from children. Eur J Clin Microbiol Infect Dis.

[20] Gumbo T, Louie A, Deziel MR, Liu W, Parsons LM, Salfinger M, Drusano GL (2007). Concentration-dependent *Mycobacterium tuberculosis* killing and prevention of resistance by rifampin. Antimicrob Agents Chemother.

